# Processing of Pre-microRNAs by the Dicer-1–Loquacious Complex in *Drosophila* Cells

**DOI:** 10.1371/journal.pbio.0030235

**Published:** 2005-05-24

**Authors:** Kuniaki Saito, Akira Ishizuka, Haruhiko Siomi, Mikiko C Siomi

**Affiliations:** **1**Institute for Genome Research, University of Tokushima, Kuramoto, Tokushima, Japan; Oregon State UniversityUnited States of America

## Abstract

microRNAs (miRNAs) are a large family of 21- to 22-nucleotide non-coding RNAs that interact with target mRNAs at specific sites to induce cleavage of the message or inhibit translation. miRNAs are excised in a stepwise process from primary miRNA (pri-miRNA) transcripts. The Drosha-Pasha/DGCR8 complex in the nucleus cleaves pri-miRNAs to release hairpin-shaped precursor miRNAs (pre-miRNAs). These pre-miRNAs are then exported to the cytoplasm and further processed by Dicer to mature miRNAs. Here we show that *Drosophila* Dicer-1 interacts with Loquacious, a double-stranded RNA-binding domain protein. Depletion of Loquacious results in pre-miRNA accumulation in *Drosophila* S2 cells, as is the case for depletion of Dicer-1. Immuno-affinity purification experiments revealed that along with Dicer-1, Loquacious resides in a functional pre-miRNA processing complex, and stimulates and directs the specific pre-miRNA processing activity. These results support a model in which Loquacious mediates miRNA biogenesis and, thereby, the expression of genes regulated by miRNAs.

## Abstract 2

This and an accompanying paper by Förstemann et al. identify Loquacious, which encodes a double-stranded RNA binding domain protein, and partners with Dicer-1 in the processing of microRNAs.

## Introduction

microRNAs (miRNAs) act as RNA guides by binding to complementary sites on target mRNAs to regulate gene expression at the post-transcriptional level in plants and animals [1−12], much as small interfering RNAs (siRNAs) do in the RNA interference (RNAi) pathway [13−15]. The expression of miRNAs is often developmentally regulated in a tissue-specific manner, suggesting an important role for miRNAs in the regulation of endogenous gene expression [16–30]. The importance of miRNAs for development is also highlighted by a recent computer-based analysis that predicted nearly a thousand miRNA genes in the human genome [31]. Furthermore, recent studies have revealed that miRNAs regulate a large fraction of the protein-coding genes [32–34].

miRNAs are transcribed as long primary miRNA (pri-miRNA) transcripts by RNA polymerase II [35]. miRNA maturation begins with cleavage of the pri-miRNAs by the nuclear RNase III Drosha [36–38] to release approximately 70-nucleotide hairpin-shaped structures, called precursor miRNAs (pre-miRNAs). Pre-miRNAs are then exported to the cytoplasm by the protein Exportin 5, which recognizes the two-nucleotide 3′ overhang that is a signature of RNase III-mediated cleavage [39–41]. In the cytoplasm, pre-miRNAs are subsequently cleaved by a second RNase III enzyme, Dicer, into approximately 22-nucleotide miRNA duplexes, with an end structure characteristic of RNase III cleavage [42–44]. Only one of the two strands is predominantly transferred to the RNA-induced silencing complex (RISC) [45], which mediates either cleavage of the target mRNA or translation silencing, depending on the complementarity of the target [46] by a mechanism that remains unclear [47].

There is a growing list of double-stranded RNA (dsRNA)-binding proteins that play important yet distinct roles in the RNAi pathway [48]. Both Drosha and Dicer contain dsRNA-binding domains (dsRBDs). Drosha requires a dsRNA-binding protein partner known as Pasha in flies and *Caenorhabditis elegans,* and its ortholog DGCR8 in mammals to convert pri-miRNAs to pre-miRNAs [49–52]. In plants, the predominantly nuclear Dicer-like-1, equipped with two dsRBDs, is thought to catalyze both pri-miRNA and pre-miRNA processing [53,54]. The HYL1 protein, which also contains a tandem dsRBD, is required for miRNA accumulation and may play the same molecular role as Pasha/DGCR8 for Dicer-like-1 in plants [55,56]. In *Drosophila,* Dicer-2 is required for production of siRNAs [57,58], and forms a heterodimeric complex with the dsRNA-binding protein R2D2, which is required for its function in RISC assembly, although Dicer-2 alone suffices to convert long dsRNA into siRNAs [59]. *Drosophila* Dicer-1 is associated with the processing of pre-miRNAs [58,60]. However, if there is a dsRNA-binding protein partner for Dicer-1, it has not been identified.

Here, we show that *Drosophila* Dicer-1 interacts with the dsRBD protein Loquacious (Loqs). Depletion of Loqs results in accumulation of pre-miRNAs in *Drosophila* S2 cells. Loqs is predominantly cytoplasmic and is conserved in mammals. Immuno-affinity purification experiments, together with the use of recombinant Loqs, reveal that along with Dicer-1, Loqs resides in a functional pre-miRNA processing complex, and stimulates and directs specific pre-miRNA processing activity. These results support a model in which Loqs mediates miRNA biogenesis and, thereby, the expression of genes regulated by miRNAs.

## Results

We have used RNAi-based reverse-genetic methods [61] to screen a list of *Drosophila* dsRBD proteins [62] for a protein(s) that has an effect on miRNA biogenesis in *Drosophila* S2 cells and found a novel protein equipped with three dsRBDs (two canonical dsRBDs at the N-terminal half, and one non-canonical dsRBD at the C-terminal), originally dubbed CG6866 (candidate gene 6866), which has a role in pre-miRNA processing (data presented below). This protein bears high similarity to R2D2 and to the *C. elegans* RNAi protein RDE-4 (Figure 1), both of which contain dsRBDs and interact with Dicer [59,63]. Thus the sequence data show that CG6866 is a paralog of R2D2. A parallel study presents genetic evidence that several types of silencing are lost in CG6866 mutant flies (Förstemann K, et al. DOI: 10.1371/journal.pbio.0030236). Therefore, CG6866 was designated as Loquacious (“very talkative”).

**Figure 1:**
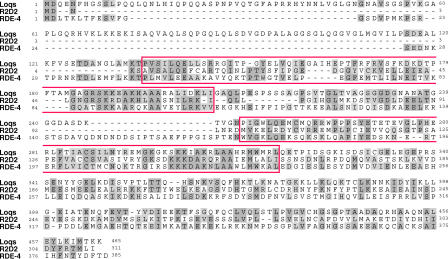
Loqs/CG6866 Is a Paralog of R2D2 A protein sequence alignment of Loqs, R2D2, and RDE-4 (*C. elegans*). The two canonical dsRBDs are boxed. Conserved residues are shaded in gray. It is noted that the C-terminal region of Loqs contains a non-canonical dsRBD (see Figure 8).

### Depletion of Loqs and Dicer-1 by RNAi Resultsin Pre-miRNA Accumulation

Dicer-1 has been shown to be the pre-miRNA processing factor in *Drosophila* [58]. We have previously shown that depletion of Dicer-1 by RNAi resulted in a marked accumulation of pre-*miR-bantam* (pre-*miR-ban*) [60]. Depletion of Loqs by RNAi resulted in a similar effect to Dicer-1 depletion for *miR-ban* (Figure 2A). Loqs dsRNAs caused the suppression of Loqs mRNA (Figure 2B). RNAi against Loqs does not appear to affect Dicer-1 protein levels (lower panel in Figure 2B), suggesting that the observed pre-miRNA accumulation in Loqs-depleted cells is not simply due to destabilizing Dicer-1. Similar effects on *miR-8* were seen in Dicer-1- and Loqs-depleted S2 cells (Figure 2C). Depletion of Dicer-2 and R2D2, which form the enzyme complex predominantly responsible for generating siRNAs from long dsRNA [59], had no significant effect on pre-miRNA processing (Figure 2A and 2C). These results show that along with Dicer-1, Loqs is essential for efficient pre-miRNA processing in vivo.

**Figure 2:**
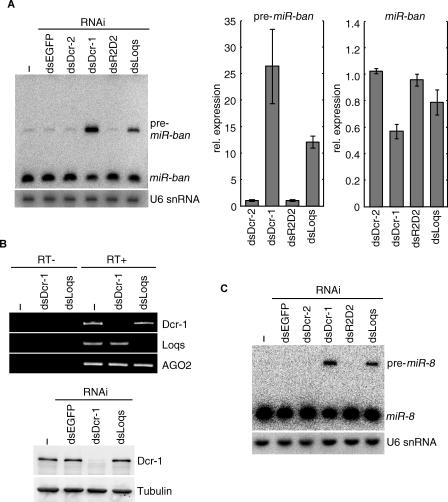
Depletion of Loqs and Dicer-1 Causes Pre-miRNA Accumulation (A) dsRNAs of Loqs, Dicer-1 (Dcr-1), Dicer-2 (Dcr-2), R2D2, or EGFP were introduced to S2 cells by soaking. Total RNA was isolated 4 d after initial exposure of S2 cells to the indicated target genes. The RNA was separated on a 12% denaturing polyacrylamide gel, transferred to a nylon membrane, and probed with 5′-radiolabeled *miR-bantam* *(miR-ban)* antisense oligodeoxynucleotide (top panel) and re-probed for U6 snRNA (bottom panel). After normalization for loading, the relative ratios for pre-miRNA (pre-*miR-ban*) (left panel) and mature miRNA *(miR-ban)* (right panel) were calculated and normalized to EGFP dsRNA experiments. Depletion of Dicer-1 (Dcr-1) by RNAi resulted in a marked accumulation of pre-*miR-ban* and a modest reduction in levels of mature *miR-ban*. (B) Upper panel: Analysis of transcript levels using RT-PCR for Loqs and Dicer-1 (Dcr-1) after treatment of *Drosophila* S2 cells with dsRNAs against each protein. AGO2 was used as control. Lower panel: Loqs dsRNA did not affect Dicer-1 protein levels. dsRNAs of Loqs, Dicer-1, or EGFP were introduced to S2 cells by soaking. After 4 d, cell lysates were prepared and the levels of Dicer-1 protein were measured by Western blotting. (C) As in (A), total RNA was probed with 5′-radiolabeled *miR-8* antisense oligodeoxynucleotide (top panel) and re-probed for U6 snRNA (bottom panel).

### Loqs Associates with Dicer-1 In Vivo and In Vitro

This observation prompted us to ask if Loqs forms a complex in vivo with Dicer-1. For these studies, we simultaneously expressed Dicer-1 tagged with the Flag epitope and Loqs tagged with the myc epitope in S2 cells. We then immunoprecipitated Dicer-1 with anti-Flag antibodies, and Loqs with anti-myc antibody and then analyzed the precipitates by immunoblotting (Figure 3A). In reciprocal assays, Dicer-1 and Loqs were found to co-precipitate. Consistent with these findings that Dicer-1 and Loqs form a complex in vivo, both proteins are localized predominantly in the cytoplasm of S2 cells (Figure 3B).

**Figure 3:**
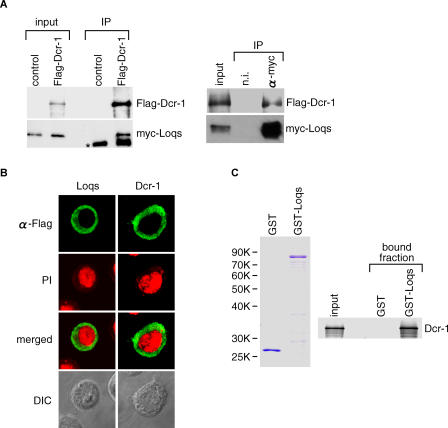
Loqs Associates with Dicer-1 (A) Loqs and Dicer-1 form a complex in vivo. Left panel: Protein extract was prepared from S2 cells that expressed both full-length myc-tagged Loqs (myc-Loqs) and Flag-tagged Dicer-1 (Flag-Dcr-1). Protein extract containing only myc-Loqs was also prepared (control). Total extracts (input) and the materials obtained after immunoprecipitation from the extracts with anti-Flag were run on an SDS-polyacrylamide gel. Western blots were prepared and immunostained with anti-myc (to detect myc-Loqs) or anti-Flag (to detect Flag–Dicer-1) antibodies. The protein band shown by an asterisk is an antibody used. Right panel: Protein extracts were prepared from S2 cells that expressed both myc-Loqs and Flag–Dicer-1, and immunoprecipitation was performed with anti-myc antibody (α-myc). Non-specific antisera were also employed as a negative control (n.i.). Flag–Dicer-1 was specifically co-immunoprecipitated with myc-Loqs. (B) Immunofluorescence using anti-Flag antibodies show that both Loqs and Dicer-1 are predominantly localized in the cytoplasm in *Drosophila* S2 cells (α-Flag). Flag–Dicer-1 (Dcr-1) and Flag–Loqs were transiently expressed in the cells by transfection. The nuclear DNA was stained with propidium iodide. A DIC image of the same field is also shown (DIC). (C) Loqs interacts with Dicer-1 in an RNase-resistant manner in vitro. ^35^S-labeled Dicer-1 was produced by an in vitro transcription and translation system in the presence of [^35^S]methionine, treated with RNaseA, and incubated with either GST–Loqs or GST itself immobilized on glutathione-Sepharose resins. After extensive washing, the bound fractions were resolved on an SDS-polyacrylamide gel and the protein labeled with ^35^S visualized by autoradiography. The Coomassie Blue stainings of GST and GST–Loqs used in this experiment are shown on the left.

We further investigated whether Loqs can bind to Dicer-1 in vitro. Dicer-1 was produced by an in vitro translation system and used in binding assays with recombinant Loqs fused to glutathione S-transferase (GST). GST–Loqs interacted with Dicer-1 even in the presence of RNase A, whereas GST itself showed no detectable binding (Figure 3C). These results demonstrate that the association of Loqs with Dicer-1 occurs both in vivo and in vitro, and that RNA molecules do not appear to mediate the association.

### Dicer-1 and Loqs Are Present in a Functional Complex That Mediates Pre-miRNA Processing

To examine the functional connection between the Dicer-1–Loqs complex and pre-miRNA processing, we investigated if depletion of Dicer-1 or Loqs had any effect on the production of mature miRNA from the precursor. We first tested if cytoplasmic lysates of S2 cells were capable of processing synthetic *Drosophila melanogaster* *let-7* precursor RNA into functional mature *let-7*. In this experiment, the synthetic *let-7* precursor RNA was converted to mature *let-7* in S2 cytoplasmic lysates (Figure 4A), as is the case in embryo lysates [60]. In in vitro RNAi assay, target RNA harboring a sequence perfectly complementary to mature *let-7* was cleaved efficiently within the *let-7* complementary sequence (Figure 4B), thus showing production of functional *let-7* in S2 cell lysates. Cytoplasmic lysates from Dicer-1- or Loqs-depleted cells were then subjected to the pre-*let-7* processing assay. Both Dicer-1 and Loqs depletion led to reductions of mature *let-7* compared with controls (Figure 4C), showing that both Dicer-1 and Loqs function in pre-miRNA processing.

**Figure 4:**
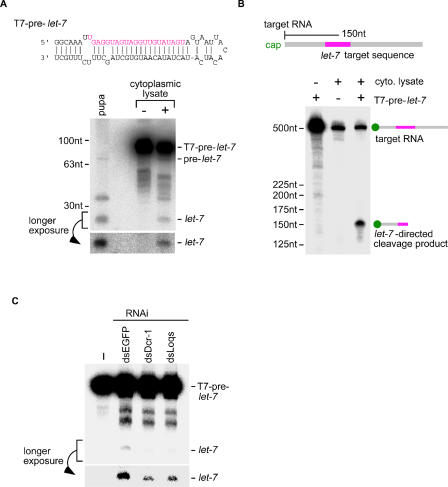
Synthetic *D. melanogaster* *let-7* Precursor Processing in the Cytoplasmic Lysate of S2 Cells (A) S2 cytoplasmic lysate is capable of processing pre-*let-7* into mature *let-7*. Pre-*let-7* (the sequence shown on the top) was transcribed with T7 RNA polymerase in vitro (T7-pre-*let-7*). After gel purification, it was incubated with S2 cytoplasmic lysate, and resultant RNA was subjected to Northern blotting using an oligodeoxynucleotide recognizing both pre-*let-7* and mature *let-7*. Total RNA prepared from pupa was also applied (pupa), which shows where endogenous pre-*let-7* (pre-*let-7*; ∼70 nucleotides) and mature *let-7* (*let-7*; 21 nucleotides) migrate on the blot. The cytoplasmic lysate (+) lane, but not (−) lane, shows a band corresponding to mature*let-7,* meaning that the synthetic T7-pre-*let-7* was processed to the matured form in the lysate. (B) Functional analysis of mature *let-7* produced from synthetic T7-pre-*let-7* in (A). Cap-labeled target RNA with *let-7* target site (∼500 nucleotides) was incubated in S2 cytoplasmic lysate with or without T7-pre-*let-7* for 3 h.*let-7*-directed cleavage product (∼150 mucleotides) was observed only when T7-pre-*let-7* was included, indicating that *let-7* produced in the lysate is functional. (C) Dicer-1- or Loqs-depleted cytoplasmic lysate has less activity to produce mature *let-7* from the precursor. Dicer-1 (Dcr-1) or Loqs was depleted from S2 cells by RNAi, and the cytoplasmic lysate was assayed for pre-*let-7* processing as in (A). "-" indicates no lysate.

We next used pre-*miR-ban* as a substrate for pre-miRNA processing assays. It was shown recently that S2 cell extracts contained pri-miRNA processing activity that cleaved pri-miRNA into an approximately 60- to 70-bp pre-miRNA precursor [49]. This processing is known to occur in the nucleus; thus pre-*miR-ban* was prepared by in vitro processing of pri-*miR-ban* incubated with S2 nuclear extracts (Figure 5A). Uniformly labeled pre-*miR-ban* was then gel-purified and used as a substrate for analysis of pre-miRNA processing. Incubation of the pre-miRNA with S2 cytoplasmic extracts resulted in the appearance of a mature 21-nucleotide *miR-ban* (Figure 5B). We then examined the requirement of Dicer-1 and Loqs in pre-*miR-ban* processing. Incubation of pre-miRNA with Dicer-1- and Loqs-depleted S2 cytoplasmic extracts resulted in a marked reduction in mature miRNA levels (Figure 5B). In contrast, depletion of Dicer-2 or R2D2 showed no measurable reduction of mature miRNA levels (Figure 5B). We then assayed the pre-miRNA processing activity of the purified complexes (both Flag–Dicer-1 and Flag–Loqs complexes). That the Flag–Loqs complex contains Dicer-1 was confirmed by immunoblotting (data not shown). Both Dicer-1 and Loqs complexes were capable of generating mature*miR-ban* from pre-*miR-ban* (Figure 5C). Several steps in the RNAi and miRNA pathways are known to require a divalent metal ion [64]. In addition, it is well known that RNase III-type enzymes require divalent metals for cleavage [65]. Flag–Dicer-1 complex was employed and the processing was performed in the presence of magnesium ions or EDTA in a buffer. As shown in Figure 5D, no pre-miRNA processing activity was detected at 10 mM EDTA. These results demonstrated that the Dicer-1–Loqs complex converts pre-miRNAs into mature miRNAs in a divalent metal ion–dependent manner.

**Figure 5:**
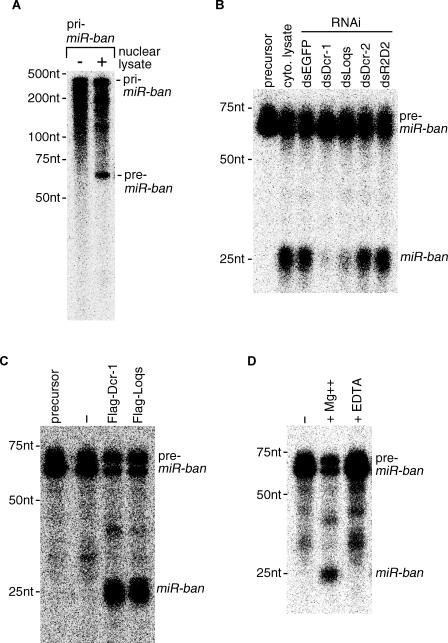
In Vitro Processing Activities of Loqs and Dicer-1 (A) Preparation of pre-*miR-ban*. Uniformly labeled pri-*miR-ban* was incubated with S2 nuclear lysate for the processing. The resultant pre-*miR-ban* fragment (pre-*miR-ban*) was gel-purified and used as a substrate in the pre-miRNA processing assays. (B) In vitro processing of pre-*miR-ban* using S2 cytoplasmic lysates. Cytoplasmic lysates were prepared 4 d after the initial exposure of S2 cells to the indicated target genes (as in Figure 2A) and used for in vitro processing. The gel-purified pre-*miR-ban* was incubated in cytoplasmic lysates for 1 h. “cyto. lysate” indicates parental S2 cytoplasmic lysate that shows activity for generating mature *miR-ban* from the gel-purified pre-*miR-ban* in (A). (C) In vitro processing of pre-*miR-ban* using immunopurified Dicer-1 and Loqs complexes. Purified Flag–Dicer-1 (Flag-Dcr-1) and Flag–Loqs complexes were incubated with pre-*miR-ban* for 2 h and tested for processing activity. “−” shows the activity of a negative control prepared from parental S2 cells. (D) The pre-miRNA processing activity of Flag–Dicer-1 complex in the presence and absence of magnesium ions. Purified Flag–Dicer-1 complex was incubated with pre-*miR-ban* with or without magnesium ions in buffers. Addition of EDTA caused the abolition of the activity.

### Loqs Stimulates and Confers upon Dicer-1 the Specific Processing of Pre-miRNAs

To further examine the requirement for Loqs in pre-miRNA processing, we purified Flag–Dicer-1 complex under a harsher condition (high salt), where Dicer-1 was stripped of most Loqs protein (Figure 6A), and used this Dicer-1 complex in pre-miRNA processing assays with or without supplement of recombinant GST–Loqs (see left panel in Figure 3C). Without any supplement, the Flag–Dicer-1 complex purified under the harsh condition showed less activity than that under mild condition (Figure 6B). Then we added GST–Loqs in the assay mixture. The addition of GST–Loqs to the Dicer-1 complex stimulated the processing of pre-miRNA (Figure 6C). GST–Loqs alone did not show any significant pre-miRNA processing activity (Figure 6C). These results show that Loqs is required for stimulating the processing of pre-miRNAs. Interestingly, we found that the Dicer-1 complex purified under the harsh condition displayed considerable siRNA-generating activity on the long dsRNA substrate in vitro (Figure 6D), although previous genetic studies have shown that Dicer-1 is not required for siRNA production [58]. The addition of GST–Loqs inhibited this effect (Figure 6D). Western blot analysis failed to show that the Dicer-1 complex used in this experiment contains Dicer-2 (right panel inFigure 6D). GST–Loqs alone showed no activity for generating siRNAs from long dsRNAs. These results suggested that Dicer-1 stripped of much of its bound Loqs processes both dsRNA and pre-miRNA substrates, but re-addition of recombinant Loqs suppresses dsRNA processing activity and enhances pre-miRNA processing activity. Our findings thus imply that much of the apparent substrate specificity of Dicer-1 in vivo results from its association with Loqs. Although very unlikely (Figure 6D), it is, however, formally possible that the Dicer-1 immunoprecipitates may contain very small amounts of Dicer-2 protein that can catalyze long dsRNA cleavage, and that addition of a large amount of dsRBD-containing Loqs may block the activity of Dicer-2 in this experiment.

**Figure 6:**
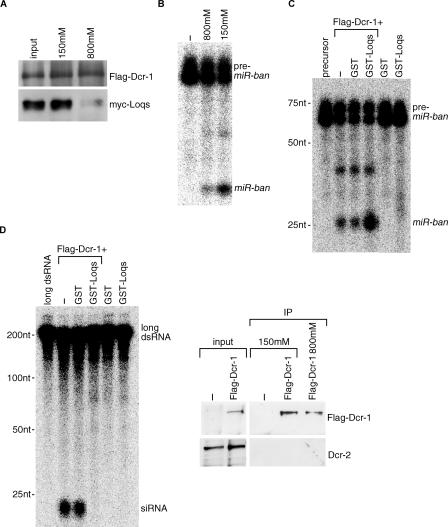
Loqs Stimulates the Specific Processing of Pre-miRNA by Dicer-1 (A) Flag–Dicer-1 complex was purified under a harsh condition. Protein extract was prepared from S2 cells that expressed both full-length myc-tagged Loqs (myc-Loqs) and Flag-tagged Dicer-1 (Flag-Dcr-1). The amounts of Loqs in Flag–Dicer-1 complexes prepared under high-salt condition and low-salt condition were examined by Western blotting using anti-myc antibody, which show that less Loqs was co-purified with Dicer-1 in the high-salt condition. (B) The miRNA processing activities of Flag–Dicer-1 (Flag-Dcr-1) complexes in (A). Flag–Dicer-1 complex containing less Loqs showed a lower activity for the processing. (C) Recombinant Loqs stimulates the in vitro processing of pre-*miR-ban* by Flag–Dicer-1 complex purified in high-salt condition. 100 ng of purified GST or GST–Loqs (see Figure 3C) were supplemented for the processing activity by Flag–Dicer-1 complex. GST–Loqs by itself does not show any pre-miRNA processing activity. (D) GST–Loqs inhibits the siRNA-generating activity of Dicer-1. Uniformly labeled long dsRNA was incubated with Flag–Dicer-1 (Flag-Dcr-1) complex purified in high-salt condition in (A) with or without GST–Loqs. The Flag–Dicer-1 complex by itself showed a considerable activity of generating siRNA from long dsRNA. Addition of GST–Loqs, but not GST, inhibited the processing. Note that the Flag–Dicer-1 (Flag-Dcr-1) complex does not contain Dicer-2 (Dcr-2), judged by Western blot analysis using anti-Dicer-2 antibodies (right panel).

### Dicer-1–Loqs Complexes Associate with Pre- and Mature miRNAs In Vivo

We examined the presence of endogenous miRNA in RNA preparations from Flag–Dicer-1 and Flag–Loqs complexes obtained from S2 cells using anti-Flag antibodies. Complexes were prepared as in Figure 3A, and RNA preparations from each complex were subjected to Northern blotting using an oligo probe recognizing both pre-*miR-ban* and mature *miR-ban*. The Dicer-1 complex contained both the pre- and mature form of *miR-ban,* and the complex seems to preferentially bind the precursor form of *miR-ban* (Figure 7A). In contrast, the precursor form of *miR-ban* was barely detectable in the Loqs complex, though it contained mature *miR-ban*. However, EDTA treatment, which inhibits pre-miRNA processing activity (see Figure 5D), resulted in an accumulation of pre-*miR-ban* in the Loqs complex (Figure 7A). This may suggest that part of Flag-tagged Loqs protein interacts with Dicer-1 or pre-miRNAs or both. Alternatively, Flag–Loqs complexes may rapidly process pre-miRNAs into mature miRNAs and, therefore, may only transiently interact with them. Nonetheless, these results suggest that Dicer-1–Loqs complexes associate with both pre- and mature miRNAs in vivo.

**Figure 7:**
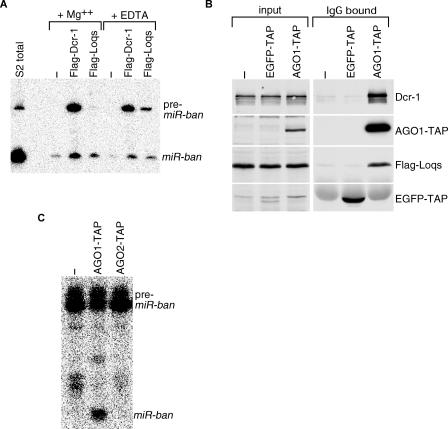
Dicer-1–Loqs Complexes Are Associated with Pre-miRNA and Mature miRNA In Vivo (A) Northern blot analyses show that Flag–Dicer-1 (Flag-Dcr-1) complex contains both pre- and mature form of *miR-ban*. Notably, the precursor form of *miR-ban* was accumulated in the Dicer-1 complex. In the case of the Loqs complex, the precursor apparently accumulated within the complex when the Flag–Loqs complex was prepared in the presence of EDTA that was shown to inhibit the pre-miRNA processing activity in Figure 5D. (B) AGO1 associates with Dicer-1 and Loqs. IgG-bound fractions prepared from S2 cells expressing AGO1–TAP, EGFP–TAP, or the parental S2 cells (−), were subjected to Western blotting using antibodies against Dicer-1, AGO1, and Flag (for Loqs). Dicer-1 is not present in AGO2-associated complex. (C) In vitro processing of pre-*miR-ban* using affinity-purified AGO1 complexes. Purified AGO1–TAP or AGO2–TAP complexes were incubated with pre-*miR-ban* (precursor) and tested for processing activity. “−” shows the activity of a negative control prepared from parental S2 cells.

### An AGO1-Associated Complex Contains Dicer-1 and Loqs, and Is Capable of Pre-miRNA Processing

We have previously shown that Argonaute protein AGO1 is required for stable production of mature miRNAs and associates with Dicer-1 [60]. Thus, we sought to ascertain if Loqs was also present in an AGO1-associated complex, and if so, if the AGO1 complex was capable of processing pre-miRNA in vitro. We simultaneously expressed Flag–Loqs and AGO1 tagged with TAP in S2 cells and purified the AGO1–TAP complex through immunoglobulin G (IgG) bead-binding. The IgG bound was then subjected to Western blot analysis using anti-Dicer-1, anti-AGO1, or anti-Flag (for Loqs detection) antibodies. Not only Dicer-1 but also Loqs was detected in the AGO1 complex (Figure 7B). These results indicated that all three proteins are present in the same complex, although they cannot exclude the possibility that there is one complex that contains AGO1 and Dicer-1 but not Loqs, and another complex that contains AGO1 and Loqs but not Dicer-1. The pre-miRNA processing activity of the AGO1 complex was then examined. As in Figure 5, pre-*miR-ban* was utilized as a substrate. The AGO1 complex was able to efficiently process pre-*miR-ban* into the mature form (Figure 7C). In contrast, another Argonaute protein AGO2-associated complex showed no such activity, which is consistent with our previous finding that the AGO2-associated complex does not contain Dicer-1 [60]. Considered together, these results showed that Dicer-1 and Loqs form a functional complex that mediates the genesis of mature miRNAs from pre-miRNAs, and suggested that the resultant mature miRNAs are loaded onto an AGO1-associated complex, which probably is miRNA-associated RISC [60], through specific interaction of AGO1 with Dicer-1 and Loqs.

## Discussion

Our results indicate that Loqs and Dicer-1 form a complex that converts pre-miRNAs into mature miRNAs; so how do they act together in pre-miRNA processing? Sequence comparison reveals that Loqs is a paralog of R2D2 (see Figure 1). Therefore, Loqs may play the molecular role of R2D2 for Dicer-1. R2D2 forms a stable heterodimeric complex with Dicer-2, while either protein alone seems to be unstable in vivo [59]. In the absence of R2D2, Dicer-2 is still capable of efficiently processing long dsRNA into siRNAs. Therefore, the siRNA generating activity of Dicer-2 is not dependent upon R2D2. However, the resultant siRNAs are not effectively channeled into RISC in the absence of R2D2. The Dicer-2–R2D2 complex, but not Dicer-2 alone, binds to siRNA, which indicates that siRNA binding by the heterodimer is important for RISC entry [59,66]. In the case of Loqs, this protein alone is not capable of converting pre-miRNAs into mature miRNAs, but it clearly stimulates and directs the specific pre-miRNA processing activity of Dicer-1. Furthermore, knocking down Loqs markedly reduced the pre-miRNA processing activity in cytoplasmic lysates in vitro (see Figures 4C and 5B), but did not cause a significant reduction of the level of Dicer-1 protein (see Figure 2B); implying that Dicer-1 may largely depend on Loqs for its pre-miRNA processing activity. Thus, the molecular role of Loqs for Dicer-1 is not simply similar to that of R2D2 for Dicer-2.

It can be envisioned that Loqs may have one of several roles in pre-miRNA processing. Dicer-1 contains only one dsRBD, which may not be sufficient for strong interaction with and/or specific recognition of the pre-miRNA substrate (see Figure 6C and 6D). Loqs, containing three dsRBDs with no other identifiable domains being apparent, could provide the additional RNA-binding modules required for specific recognition of the pre-miRNA, and thereby stabilize pre-miRNA binding for Dicer-1. Loqs could also organize binding of Dicer-1 on the pre-miRNA, contributing to the specific positioning of the Dicer-1 cleavage site. Alternatively, since dsRBDs are known to not only bind dsRNAs but also mediate protein–protein interactions [67], Loqs may directly bind Dicer-1 through its dsRBDs. This protein–protein interaction may trigger a conformational change of Dicer-1 that facilitates either the formation of an intramolecular dimer of its two RNase III domains [50,68], which creates a pair of catalytic sites, or the handover of the Dicer-1 cleaved mature miRNAs to the RISC.

Sequence analysis revealed that protein activator of protein kinase dsRNA dependent (PKR) (PACT) [69] and HIV TAR RNA binding protein (TRBP) [70] in mammals bear 34% identity to Loqs, and share a highly similar domain structure with it (Figure 8). Both PACT and TRBP are thought to play a role in the regulation of translation through modulating PKR that also contains two dsRBDs [71–73]. PACT interacts with PKR and enhances the autophosphorylation of PKR [67], which in turn, phosphorylates the α subunit of eukaryotic translation initiation factor 2 (eIF2α) and leads to an inhibition of mRNA translation in response to viral infection and other stimuli. TRBP prevents PKR-mediated inhibition of protein synthesis through binding to PKR [74]. Considered together, it will be important to find out Loqs' partners other than Dicer-1 for possible involvement of Loqs in miRNA-mediated translational regulation in *Drosophila*.

**Figure 8:**
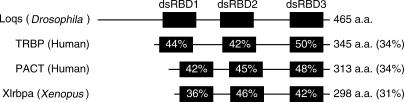
Domain Structure of Loqs and Its*Homo sapiens* and *Xenopus* Homologs The dsRNA-binding motif (dsRBD) is indicated as a black box. These proteins contain three putative dsRBDs. Loqs shares ∼34% amino acid identity with TRBP and PACT and ∼31% identity with Xlrbpa (*Xenopus laevis* RNA-binding protein A). Sequence comparison between Loqs and its human and *Xenopus* homologs also showed a higher degree of amino acid conservation in dsRBDs including C-terminal non-canonical dsRBDs. The*Xenopus* homolog, Xlrbpa, of TRBP/PACT has been found to associate with ribosomes in the cytoplasm [77], as is the case for many RNAi factors including miRNAs [47,78−82].

## Materials and Methods

### RNAi

dsRNAs were introduced to S2 cells by soaking essentially as described [75]. Briefly, approximately 5 × 10^6^ cells were soaked in 1 ml of serum-free medium containing 15 μg of dsRNA for 30 min at room temperature followed by addition of 2 ml of the medium containing 15% serum, 3 mM glutamine, and penicillin-streptomycin. After 4 d, cells were harvested and subjected to total RNA preparation for Northern blot analysis, or cytoplasmic lysate preparation for in vitro processing assays. dsRNAs used in RNAi were: double-stranded RNA for enhanced green fluorescent protein (EGFP), homologous to nucleotides 11–717 of the EGFP coding sequence; dsDcr-2, 4091–4888 of the Dicer-2 coding; dsR2D2, 1–936; dsDcr-1, 10–950 of the Dicer-2 coding; dsLoqs, 330-1342.

### Northern blot analysis

Total RNA was isolated from S2 cells with ISOGEN (Nippon Gene, Toyama, Japan). 20 μg of total RNA was separated on 12% acrylamide-denaturing gel and transferred onto Hybond-N+ membrane (Amersham Bioscience, Little Chalfont, United Kingdom). After UV-crosslinking, the hybridization was performed at 42 °C in 0.2 M sodium phosphate (pH 7.2), 7 % SDS, and 1 mM EDTA with end-labeled antisense oligodeoxynucleotide, and washed at 42 °C in 2× saline sodium citrate and 0.1% SDS. Oligodeoxynucleotides used as probes were: bantam, 5′- CAGCTTTCAAAATGATCTCAC-3′; miR-8, 5′- GACATCTTTACCTGACAGTATTA-3′; U6 snRNA, 5′- GGGCCATGCTAATCTTCTCTGTA-3′; and let-7, 5′- AACTATACAACCTACTACCTCA-3.′ The blots were exposed on BAS-MS2040 imaging plates, and signals were quantified using BAS-2500 (Fuji, Tokyo, Japan).

### RT-PCR analysis

One μg of total RNA was used for the first-strand cDNA synthesis with Stratascript RT and random primers (Stratagene, La Jolla, California, United States). Sequences of the oligonucleotide primers for RT-PCR were: Dicer-1, 5′- ACCAATGTACTGCGTTTGCA and 5′- GTTTGCTGATCACAGAACTTAACGTT; Loqs, 5′- ATGGACCAGGAGAATTTCCACGG-3′ and 5′- CTACTTCTTGGTCATGATCTTCAAGTAC-3′; and AGO2, 5′- GCACAAGTGTGCGGTCTTGTATT-3′ and 5′- GTGAACTGCTTAATGCATTG-3′.

### Immunofluorescence analysis

Immunofluorescence analysis was performed by fixing S2 cells with 2% formaldehyde for 15 min. Cells were permeabilized using 0.1% Triton X-100. Flag-tagged proteins were stained for 30 min with anti-Flag M2 (1:1,000 dilution) antibody (Sigma, St. Louis, Missouri, United States). After extensive wash in PBS, cells were treated with 100 μg/ml RNaseA for 30 min and then stained with 0.4 μg/ml propidium iodide. Alexa-488 anti-mouse IgG was used as secondary antibody. All images were collected using a Zeiss (Oberkochen, Germany) LSM510 laser scanning microscope.

### Immunoprecipitation of Flag–Dicer-1, Flag–Loqs, and myc-Loqs

S2 cell lines, stably expressed 3× Flag-tagged Dicer-1 or Loqs, or myc-tagged-Loqs under the control of metallothionein promoter (originally from pRmHa-3 vector), were established. The expression of each protein was induced by adding copper ions into the medium. After overnight incubation, the whole cell extract was prepared in Buffer A (30 mM HEPES pH 7.4, 150 mM KOAc, 2 mM MgOAc, 5 mM DTT, 2 μg/ml Leupeptin, 2 μg/ml Pepstatin, 0.5% Aprotinin) containing 0.1% NP-40 by sonication, and followed by centrifugation. Flag–Dicer-1 and Flag–Loqs were bound to anti-Flag M2 agarose beads at 4 °C for 1 h. Immunoprecipitated proteins were then recovered with 2× SDS sample buffer or Elution buffer (Buffer A containing 400 μg/ml of 3× Flag peptides, 10% glycerol, and 100 mM KOAc). For Northern blot analysis of RNAs co-purified with Flag–Dicer-1 or Flag–Loqs, immunoprecipitates on beads were treated with ISOGEN and subjected to RNA purification.

### Protein–protein interaction assays

To produce [^35^S]methionine-labeled proteins by a TNT in vitro transcription and translation kit (Promega, Madison, Wisconsin, United States), the Dicer-1 cDNA was inserted into an expression vector, pET-28 (Novagen, Madison, Wisconsin, United States). GST pull-down assays were carried out using GST–Loqs and GST itself that were bound to glutathione Sepharose 4B resins (Amersham Biosciences) in Buffer A containing 0.1% NP-40. After incubation with TNT products and extensive washing, the bound proteins were separated by SDS-PAGE. RNaseA treatment was carried out by adding the enzyme to the binding mixture. To produce GST fusion protein, Loqs cDNA was subcloned into a pGEX-5X expression vector (Amersham Biosciences). The fusion proteins, as well as GST itself, were induced and purified as described by the manufacturer.

### Preparation of nuclear and cytoplasmic lysate for in vitro processing assays

S2 cells were suspended at approximately 1 × 10^8^ cells/ml into Hypotonic buffer (Buffer A without KOAc) and lysed by passing through a 30G needle. After centrifugation at 500 × g for 20 min, the supernatant and the precipitate were separated. The supernatant was centrifuged to obtain the supernatant as a cytoplasmic lysate. The pellet was washed twice with Hypotonic buffer and lysed by sonication in Buffer A containing 100 mM KOAc and 20% glycerol, followed by centrifugation to obtain the supernatant as a nuclear lysate. Total protein concentration in each lysate was determined with Protein assay (Bio-Rad, Hercules, California, United States) and adjusted to be equal.

### Preparation of pre-*miR-ban*

A DNA fragment coding pri-*miR-ban* was obtained from PCR reaction (primers used are: 5′- CGCTCAGATGCAGATGTTGTTGAT-3′ and 5′- GATCGGTCGGCATAAG TTCAAAGC-3′) and cloned into the SmaI site of pBluescriptSK vector in the same direction with the T3 promoter. The plasmid was digested with ClaI, gel-isolated, and used as a template for in vitro transcription reaction with MEGAscript T3 Kit (Ambion, Austin, Texas, United States) in the presence of [α^32^P]GTP. In vitro processing reaction of pri-miRNAs was performed with some modifications to previously reported method [36]. Briefly, in 1 ml reaction, 10 mM creatine phosphate, 0.5 mM ATP, 30 μg/ml creatine kinase, 0.1 U/ul RNasin, 0.1 μg yeast RNA, and 500 μl nuclear lysate were added, and pri-*miR-bantam* in 0.5× Buffer A with 100 mM KOAc was further added to the mixture. After 2 h incubation at 26 °C, RNAs were purified with ISOGEN LS (Nippon Gene) and separated on 7.5% acrylamide denaturing gel, from which pre-*miR-ban* (about 60 nucleotides in length) was recovered.

### In Vitro pre-miRNA processing assays

The condition used for in vitro pre-miRNA processing with cytoplasmic lysates was the same as that for the in vitro pri-*miR-bantam* processing. Cytoplasmic lysate used in this assay was 5 μl in a 10-μl reaction. For processing assays with purified complexes, immuno-purified Flag–Dicer-1 or Flag–Loqs was used instead of crude cytoplasmic lysate and the final concentration of buffer adjusted. For Mg^++^-depletion assay, 10 mM EDTA was added instead of Mg^++^. For the processing by Flag–Dicer-1, high-salt purified (800 mM KOAc) Flag–Dicer-1 was added in the presence or absence of bacterially produced GST–Loqs and the final concentration of buffer adjusted.

### In Vitro cleavage assay

Preparation of cap-labeled *ftz* RNA with a *let-7* target site and RNAi reaction were carried out essentially as described [60]. In brief, 10^4^ cpm of cap-labeled *let-7* target RNA was incubated with 200 nM in vitro transcribed pre-*let-7* RNA in Buffer A containing 100 mM KOAc, 10 mM creatine phosphate, 0.5 mM ATP, 30 μg/ml creatine kinase, and 0.1 U/ul RNasin. Reactions were allowed to proceed for 3 h at 26 °C. Cleavage products of the RNAi reaction were analyzed by electrophoresis on 4% denaturing polyacrylamide gels.

### TAP purification

The expression of AGO1–TAP or AGO2–TAP in S2 cells was induced by adding copper ion into the medium [60]. After overnight incubation, the cytoplasmic lysate was prepared in a Buffer A containing 150 mM KOAc. AGO1–TAP or AGO2–TAP and associated materials to the TAP-tagged fusion protein were bound to IgG Sepharose (Amersham Biosciences). Bound proteins on IgG beads were directly used for in vitro pre-miRNA processing assay, or eluted with SDS sample buffer for Western blotting analysis. The polyclonal antibodies against AGO1 were a kind gift from T. Uemura (Kyoto University) [76]. The anti-Dicer-1 antibody (AB4735) was purchased from Abcam (Cambridge, United Kingdom).

## Supporting Information

### Accession Numbers

The GenBank (http://www.ncbi.nih.gov/Genbank/) accession numbers for the genes and gene products discussed in this paper are: *ago-1* (NM_079010), *ago-2* (NM_140518), *dicer-1* (NM_079729), *dicer-2* (NM_079054), *loqs/cg6866* (NM_135802), and *r2d2* (NM_135308).

The Rfam (http://www.sanger.ac.uk/Software/Rfam/mirna/index.shtml) accession numbers for the genes and gene products discussed in this paper are: *bantam* (MI0000387), *let-7* (MI0000416), and *miR-8* (MI0000128).

## Abbreviations

dsRBD: double-stranded RNA-binding domain
dsRNA: double-stranded RNA
EGFP: enhanced green fluorescent protein
GST: glutathione S-transferase
IgG: immunoglobulin G
miRNA: microRNA
PACT: protein activator of PKR
PKR: protein kinase dsRNA dependent
pre-miRNA: precursor miRNA
pri-miRNA: primary miRNA
RISC: RNA-induced silencing complex
RNAi: RNA interference
siRNA: small interfering RNA
TRBP: TAR RNA binding protein.
